# Lattice-Mismatched van der Waals Epitaxy and Photoluminescence of Two-Dimensional Ga_*x*_In_1–*x*_Se Alloys on Si(111)

**DOI:** 10.1021/acs.cgd.6c00184

**Published:** 2026-06-30

**Authors:** Christopher P. Muzzillo, William E. McMahon, Andriy Zakutayev, Andrew G. Norman

**Affiliations:** † 53405 National Laboratory of the Rockies, 15013 Denver West Pkwy, Golden, Colorado 80401, United States

## Abstract

Ga_*x*_In_1–*x*_Se (GIS) alloys are two-dimensional (2D) layered materials with band gaps and lattice parameters of interest for many energy and electronic applications. They can be fabricated using van der Waals epitaxy, which is an emerging technique that offers unprecedented opportunities for 2D optoelectronic devices and epitaxy processes. This work has demonstrated van der Waals epitaxy of GIS alloys for the first time. Films with x compositions of 0, 0.062, 0.164, 0.680, 0.894, and 1 and tunable lattice constants were grown on Si(111) substrates, and characterized by X-ray diffraction pole figure and transmission electron microscope analysis. In spite of the lattice mismatches (InSe is 4.1% too large and GaSe is 2.8% too small), these alloys grow epitaxially, with Si(111) || GIS(001) and Si[1–10] || GIS[100] orientation. Photoluminescence was used to measure tunable band gaps in the absorber-relevant 1.3–2.0 eV range as a function of x composition and showed GIS did not degrade after capping with Se and prolonged storage. Therefore, GIS alloys exhibit a technologically advantageous combination of tunable band gap and photoluminescence with relaxed lattice parameter and rotational registry with the substrate.

## Graphical abstract

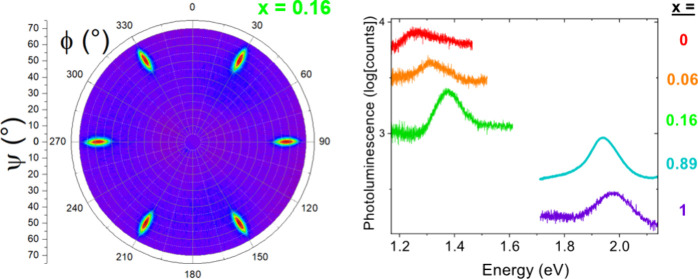

## I Introduction

Two-dimensional (2D) materials have unique properties and offer routes to completely new optoelectronic and microelectronic devices. For example, Ga_*x*_In_1–*x*_Se (GIS) alloys are 2D layered materials of interest for a wide range of applications^1–4^ including solar cells,^5–12^ photodetectors,^13–19^ light-emitting diodes,^9,20,21^ nonlinear optical applications,^22–28^ memory applications,^29^ field effect- and photo-transistors,^30–39^ neuromorphic computing,^40^ and photoelectrochemical (PEC) fuel production.^41,42^ They are also of interest for passivation of GaAs^43^ and Si,^44,45^ as buffer layers for mitigating lattice mismatch and thermal expansion mismatch for the epitaxy of GaAs on Si,^46^ and have more recently been proposed as epitaxial lift-off layers for lowering the cost of GaAs-based photovoltaics (PV).^47,48^ The Ga-rich alloys typically have the ε polytype (space group #194), hexagonal *a* lattice parameters of 3.75 to 3.775 Å, and band gaps of 1.90–2.00 eV, suitable for tandem PV or PEC device applications.^49^ The In-rich alloys have one of several polytypes resulting from different stacking sequences,^2^ hexagonal *a* lattice parameters of 3.921 to 3.985 Å, and band gaps of 1.23–1.42 eV,^49^ also suitable for PV and PEC devices. The (001) plane of hexagonal crystals has the same pattern of atoms as the (111) plane of diamond cubic and zincblende cubic crystals. Converting the hexagonal *a* lattice parameters to cubic symmetry reveals that In-rich GIS alloys are lattice-matched to commercially important 3D semiconductor substrates such as GaAs, Ge, ZnSe, etc. However, there is a miscibility gap in the InSe-GaSe tie-line^50^ for compositions 0.2 < x < 0.9.^49^

The epitaxial growth of 2D material films, like InSe, GaSe, or GIS alloys, is inherently different from traditional epitaxy of 3D materials. For the case of van der Waals epitaxy of a 2D epilayer on a 3D substrate, the substrate’s dangling bonds lead to a ‘quasi van der Waals’ interface.^51^ As the substrate/epilayer interaction is weaker than the traditional covalent 3D/3D case, unprecedented levels of lattice mismatch can be realized, and the weak bonding allows mechanical exfoliation for lift off of layer structures grown on top of 2D material buffer layers.^51^ However, the van der Waals interface also leads to unexpected relationships between the substrate and epilayer,^52^ so experimental demonstration of epitaxy for particular crystals is necessary. Currently, as far as we are aware, there are no reports of direct epitaxial growth of any ternary GIS alloys on Si.

Epitaxy of GaSe was first demonstrated on GaAs(111)­B,^43^ then on As- and H-terminated Si(111),^45,46^ Si(111),^53^ Si(100),^54^ Si(110),^54^ and more recently on α-Al_2_O_3_(001)^55,56^ and GaN(001).^56^ InSe epitaxy has been reported on mica,^57^ Si(111),^58,59^ H-terminated Si(111),^60,61^ Si(100),^62,63^ as well as GaAs(111)^64,65^ and Se-terminated GaAs(001).^66^ The growth of InSe onto GaSe films^64,67,68^ and bulk crystals^20,69–71^ can help to achieve phase-pure InSe epitaxy, which sometimes results in Ga–In intermixing, and thin GIS alloy layers at the interface.^64,67^ The In–Se phase diagram has 9 compounds^72^ (cf. three compounds for Ga–Se^73^), which may be responsible for the greater difficulty^64,67^ in achieving phase-pure epitaxy of InSe, relative to GaSe. Recent studies investigated the parameter space for InSe epitaxy on Si, for which phase growth depends sensitively on temperature and In-to-Se flux ratio.^59,62^ The goal of this work is to extend these reports on the GaSe and InSe endmembers and initiate research into the epitaxial growth behavior of this technologically important GIS alloy system.

Here, we report on GIS alloys across the composition range grown epitaxially on Si(111) and some of their structural and optoelectronic properties. The Ga_*x*_In_1–*x*_Se films with x ranging from 0 to 1 have been grown on Si(111) substrates and characterized by X-ray diffraction (XRD), transmission electron microscopy (TEM), and photoluminescence (PL). GIS alloys grew epitaxially with Si(111) || GIS(001) and Si[1–10] || GIS[100] orientation, despite the +4.1% and −2.8% lattice-mismatch for InSe and GaSe, respectively. The PL indicates tunable band gaps in the absorber-relevant 1.3–2.0 eV range and show that Se-encapsulated GIS alloys are stable even after prolonged storage. The relaxed requirements for the lattice parameter and rotational registry with the substrate make GIS alloys promising for technological applications such as PV and PEC absorbers for tandem devices and as 2D buffer and epitaxial lift-off layers on a variety of single-crystal substrates for further semiconductor material growth.

## II Experimental Methods

Molecular beam epitaxy (MBE) was performed at a rate of ∼2 μm/h and a base pressure of ≤2 × 10^–8^ Torr. Elemental fluxes were controlled by setting Knudsen source temperature, calibrating with in situ electron impact emission spectroscopy, profilometry, and X-ray fluorescence (XRF). The correlation between deposition rate by profilometry and source temperature for Se and In is shown in Figure S1. Lines fit to these data were used to calculate source temperature set points. Ga rates and set points were determined from XRF Ga/(Ga+In), or x, of the final films (Figure S2). XRF using a Fischerscope XUV was done under vacuum (pressure below 400 mTorr) with a Rh anode at 50 kV and a 10 μm Ni filter. For each sample, XRF was collected for 60 s at 10 spots, resulting in standard deviations of roughly 1 atomic-%. The Se source was a tank with a needle valve and cracker, where surfaces in contact with Se were fabricated with corrosion-resistant Ti.

Undoped Si(111) wafers with 2 in diameters and resistivity > 10^3^ Ω cm were used as the substrates. The Si wafers were ‘RCA cleaned’, with the final oxide etch delayed until just before loading into the vacuum (details are in the Supporting Information).^74^ After H desorption,^74^ the substrates were ramped at 30 °C/min to 570 °C, where they were held for 30 min to stabilize the temperature before growth. The Se shutter was opened 5 s before the Ga and/or In shutters to initiate growth. The substrate was heated with a SiC filament and rotated at 30 rpm to ensure temperature and source flux uniformity. A thermocouple near the substrate controlled the heater power, while a pyrometer measured actual substrate temperature. All shutters were closed simultaneously to end growth, followed by immediate substrate cooling at 17 °C/min. However, the Se shutter did not eliminate Se flux, so the Se temperature was also ramped down at 2 °C/min at the end of growth, which led to a ∼200 nm thick Se capping film.

X-ray diffraction (XRD) was performed on a Rigaku Dmax diffractometer with automatic specimen alignment and a rotating Cu anode at 40 kV and 250 mA producing X-rays with line focus geometry. The automated alignment routine iterated z-axis height adjustment to 50% beam intensity followed by tilt (ω) correction to maximize intensity. Sample alignment was confirmed via the substrate Si(111) peak at 28.44° 2θ. To minimize Si substrate peaks, which obscured some GIS peaks, the Si(111) peak intensity was maximized in φ; then φ was rotated by 90° (Figure S3). Symmetric XRD used focusing (Bragg–Brentano) geometry and a bent monochromator, while pole figures and grazing incidence XRD (GIXRD) used parallel beam geometry with a flat monochromator. Pole figures fix 2θ and ω at the calculated Bragg peak maximum then scan ψ from 0° to 75° in 2.5° steps and φ from 0° to 360° in 0.1° steps.

Spectrally resolved PL was collected at room temperature on the samples that were stored in an N_2_ flow box for 8 years. These measurements used a 50× lens, 0.35 numerical aperture, and 633 nm laser excitation at 0.43 mW with a spot size of 40 μm × 65 μm (5.3 × 10^19^ photons/cm^2^ s). A 532 nm laser at 1.0 mW (1.0 × 10^20^ photons/cm^2^ s) was used for PL on the wider band gap samples. Hall measurements were performed but were unreliable because Ga_*x*_In_1–*x*_Se made electrical contact to the Si substrates, unlike a recent report on InSe.^62^ Cross-sectional samples for transmission electron microscopy (TEM) were prepared using a conventional focused ion beam (FIB) lift out technique and examined in an FEI G^2^30 SuperTwin TEM operated at 300 kV.

## III Results

### A GIS Tunable Structure and Band Gap

Ga_*x*_In_1–*x*_Se (GIS) alloy films were grown on RCA-cleaned Si(111) at 570 °C after desorbing H from the substrate’s surface. As mentioned, phase-pure InSe epitaxy has historically been difficult,^64,67^ so the growth conditions were optimized for γ-InSe and then extended to GIS alloys. The optimal Se/(Ga+In) flux ratio was ∼1.2, where the process window was narrow, in agreement with previous reports.^58,60,66,70,75^ Higher and lower ratios led to γ-In_2_Se_3_ and In impurities, respectively. After growth, cooling films with Se overpressure converted InSe to γ-In_2_Se_3_, so cation and anion fluxes were shuttered at the end of each growth. The Ga/(Ga+In), or x, molar flux was changed to yield an x of 0, 0.062, 0.164, 0.680, 0.894, and 1, and the final film composition was verified by XRF.

Symmetric XRD revealed the films to be (001)-oriented, with decreasing *c* lattice parameters as x was increased (Figure 1). According to a previous study,^49^ GIS has a miscibility gap for 0.2 < x < 0.9 compositions. On the other hand, line compounds with x values of 0.5,^76,77^ 0.67,^50^ and 0.75 ^78^ have been reported. Throughout this study none of the line compounds were observed, in agreement with another recent study,^9^ and it remains unclear how they relate to the thermodynamic miscibility gap for GIS alloys. When growth was performed within the miscibility gap, the films typically phase-separated into GIS phases with x values of 0 to 0.2 and 0.8 to 0.9.

**Figure 1:**
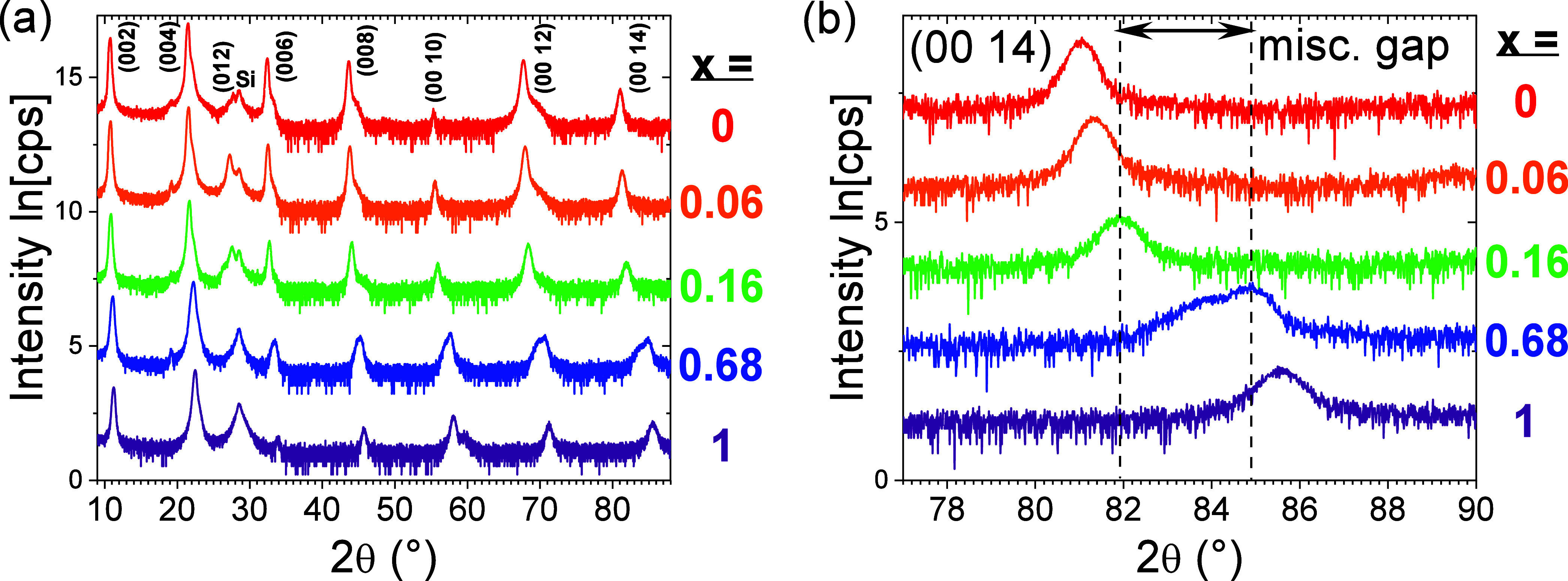
Symmetric XRD patterns for Si/GIS alloys with x compositions of 0 (top; red), 0.062 (2nd from top; orange), 0.164 (3rd from top; green), 0.680 (4th from top; blue), and 1 (bottom; purple). (a) Broad scans with GIS {001} planes labeled; Si is substrate (111). (b) Magnified (00 14) peaks, showing growth within the 0.2 < x < 0.9 miscibility gap.

However, this phase separation did not always occur: the film with an average x of 0.680 measured by XRF in Figure 1 appeared to contain GIS with an x of 0.621 and 0.829 according to their XRD lattice constants. The x = 0.621 composition was estimated from its *c* lattice parameter of 16.15 Å, along with a Vegard’s law fit to the other lattice parameters at known compositions. The metastable x = 0.621 phase (cubic *a* = 5.416 Å) is roughly lattice-matched with the Si substrate (*a* = 5.429 Å), so it may have been stabilized through the “lattice-latching” effect reported to occur during the epitaxial growth of III–V alloys^79^—although more study will be needed to establish the stability and reproducibility of this alloy composition.

The *c* lattice parameters from these films were compared with literature values for bulk GIS crystals in Figure 2. Excellent agreement with a previous study^49^ was observed. Another previous study used thermal quenching to affect In solubility in Ga-rich alloys down to an x of 0.8 (*c* of 16.12 Å),^50^ which is close to the lattice parameter for x = 0.621 measured in this work (16.15 Å), although the compositions are apparently different. This is a possible indication that certain process conditions can form metastable alloy compositions. These values are also in excellent agreement with the results recently reported for GIS single crystals grown from the liquid phase.^9^

**Figure 2:**
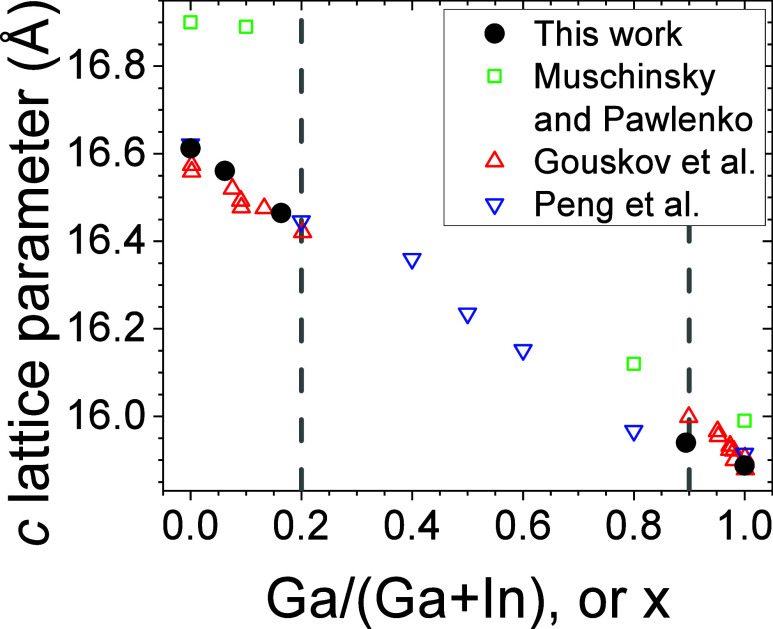
Lattice parameters, *c*, for GIS films measured in this work (black circles) and for bulk crystals from Muschinsky and Pawlenko (green squares),^50^ Gouskov et al. (red triangles),^49^ and Peng et al. (blue triangles).^9^ The gray dashed lines are the miscibility gap identified by Gouskov et al.^49^

Along with the tunable lattice constant, the samples had a tunable PL response at the expected band edges (Figure 3), even after prolonged storage in a N_2_-purged box. So, despite their documented degradation in air,^80–82^ InSe, GaSe, and Ga_*x*_In_1–*x*_Se are stable under a Se capping layer discussed below. The measured room temperature PL band gaps ranged from 1.26 to 1.98 eV, in the range relevant to solar energy conversion, and were linear with Ga/(Ga+In), in agreement with former reports of direct band gap energies for Ga_*x*_In_1–*x*_Se [Figure 3(b)].^9,39,83–85^ Films grown in the miscibility gap had PL at 1.6–1.7 eV, another indication that the forbidden alloy compositions can form, but are omitted for lack of reproducibility.

**Figure 3:**
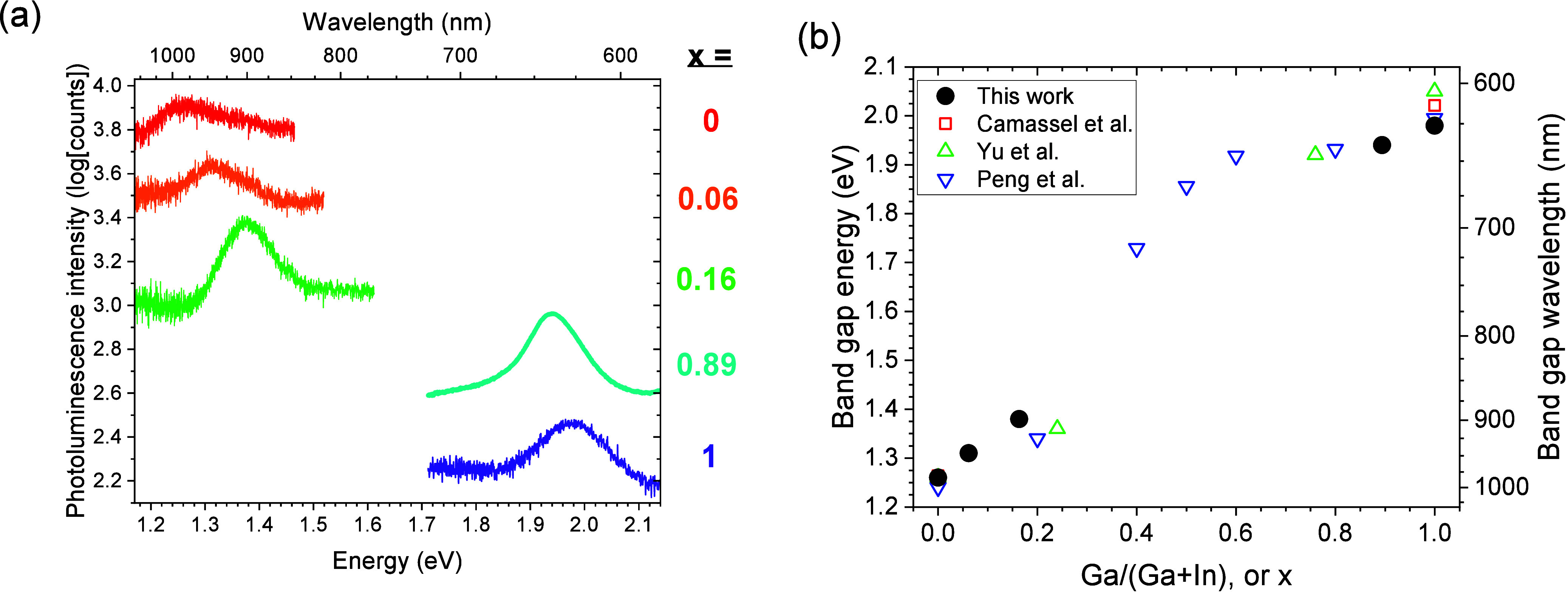
(a) Room temperature PL intensity for Ga_*x*_In_1–*x*_Se films with an x of 0 (1st red), 0.062 (2nd orange), 0.164 (3rd green), 0.894 (4th teal), and 1 (5th purple). (b) The room temperature band gap energies show linearity with Ga/(Ga+In), or x, in rough agreement with previous reports.^9,39,83,84^

Quantum confined band gap widening occurs in few-layer GaSe and InSe,^86,87^ but bulk band gaps were observed in this work’s unstrained, relatively thick GIS. Comparison of the symmetric and GIXRD peak positions and peak widths indicates that the Ga/(Ga+In) composition is uniform and single-phase throughout the film thickness (Figures S4–S8), so each film should emit at a single band gap. The films all had a single PL peak with a full width at half-maximum of 0.1 eV, so PL showed no evidence of disorder or defect-related emission. PL also showed no evidence of band gap bowing. The x = 0.894 film had stronger PL emission than the other samples, which may stem from its enhanced Se cap crystallinity (Figure S7), which is expected to be a better barrier to O_2_ and H_2_O than amorphous Se.

### B GIS Epitaxial Relation on Si

The epitaxial substrate/film relationship was explored by collecting XRD pole figures on a Si/InSe film’s {103} and {114} family of planes (Figure 4). Both pole figures exhibited 6-fold peaks, only rotated by 30°. The epitaxial relationships were therefore Si(111) || GIS(001), Si[1–10] || GIS[100], and Si[2–1–1] || GIS[110]. {103} pole figures were also collected for GIS alloy films with x values of 0.062, 0.164, 0.894, and 1 (Figures 5, S9, and S10). In all cases, the pole figures had 6 individual peaks with little disorder in the φ direction, indicating either the rhombohedral polytype (*R*3*m* space group #194; ε) or a hexagonal polytype (e.g., γ) with twinning for all x compositions. The peaks did have disorder in the ψ direction, with full-width at half-maximum values of roughly 5–10°.

**Figure 4:**
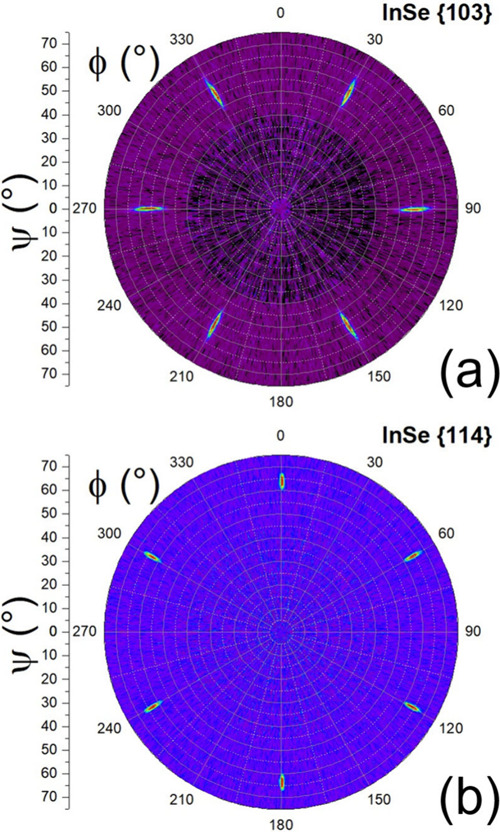
(a) {103} and (b) {114} pole figures on an epitaxial InSe film on a Si(111) substrate with φ = 0° parallel to Si[2–1–1], showing excellent rotational order in the plane of the substrate.

**Figure 5:**
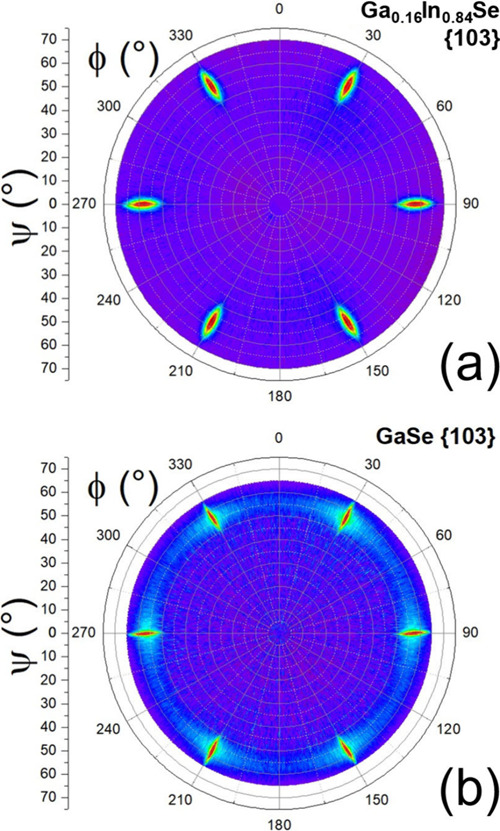
{103} pole figures on epitaxial films of (a) Ga_0.16_In_0.84_Se and (b) GaSe on Si(111) substrates with φ = 0° parallel to Si[2–1–1], showing excellent rotational order in the plane of the substrate.

The lack of rotational disorder in the plane of the substrate indicated that the films had epitaxial coherency, or registry with the substrate—despite the large lattice mismatch for all the films, ranging from 4.1% too large for InSe in Figure 4 to 2.8% too small for GaSe in Figure 5(b). The Si(111)/GIS epitaxial relationships observed in this work were in excellent agreement with those previously observed for the Si(111)/GaSe^53,88^ and Si(111)/InSe^60,75^ endmembers. The Si/GIS relationship is therefore van der Waals-like with respect to the relaxed lattice-matching requirement, but coherent or covalent-like with respect to the rotational order. These characteristics provide a technologically advantageous blend of lattice parameter/band gap flexibility and substrate/epilayer registry.

TEM was performed on Si/Ga_0.16_In_0.84_Se and Si/Ga_0.89_In_0.11_Se samples (Figures 6 and S11), which revealed that the epitaxially aligned layers were only ∼150 nm thick and were covered with a thick layer of polycrystalline GIS and amorphous Se (Figure 6(a); Figures S4–S8). Other films grown later in the study were thinner and had no GIXRD evidence of polycrystallinity at the surface (e.g., Figure S12). The (012) peaks in the x of 0, 0.062, and 0.164 films in Figure 1(a) are for space group 160. GIXRD on those films at an incident angle of 2° additionally showed polycrystalline peaks for space group 194 (Figures S4–S6), an indication of surface-segregated polycrystalline material with less (001) texture and a different polytype. On the other hand, TEM verified that the first ∼150 nm of the films was epitaxial (Figures 6(b) and S11­(a)). As the films were not strained to match the substrate’s lattice, the transition from epitaxial to polycrystalline material was not a result of strain relief. More study will be needed to elucidate a mechanism for the apparent transition in growth behavior.

**Figure 6:**
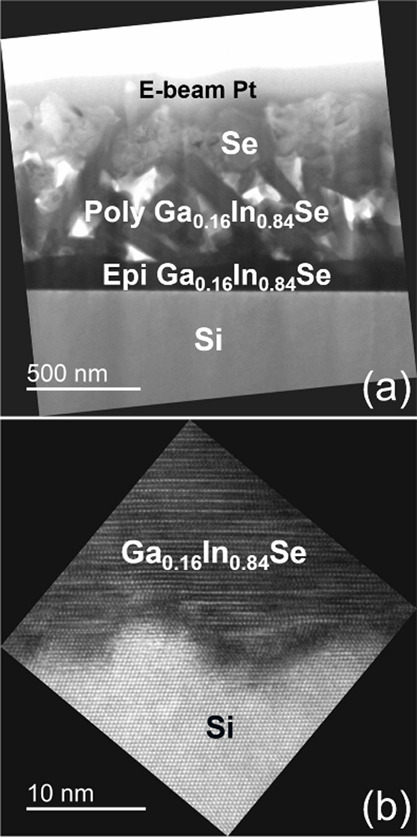
(a) Bright field TEM micrograph of a FIB-prepared cross-section from a Si/Ga_0.16_In_0.84_Se (x = 0.164) sample, showing a distinct ∼150 nm thick epitaxial layer underneath ∼500 nm of polycrystalline Ga_0.16_In_0.84_Se, and (b) HRTEM micrograph of that sample’s substrate/epilayer interface, showing lattice-nonmatched epitaxy.

## IV Discussion

The Si/GIS interface was rougher than expected (Figure 6(a)). It is speculated that background Se in the MBE reacted with the Si surface, for instance, during the pregrowth substrate temperature ramp.^89^ This could form SiSe_2_ and roughen the wafer’s surface prior to growth, which has been reported for epitaxy of GaSe^88^ and 2D Bi_2_Se_3_ ^90^ on Si(111). Previous work on GaSe found that Si(111):H, Si(111):Se, Si(111):Ga, and Ge(111):Se substrate preparation procedures all led to a covalent-bonded GaSe half-layer, either strained to the substrate or not,^91^ followed by van der Waals-bonded GaSe that is not strained to the substrate.^54,88,92–94^ Thus, SiSe_2_ can form before converting to a GaSe half-layer, allowing unstrained epitaxy to proceed despite a SiSe_2_-roughened surface and lattice-mismatch.

TEM also revealed the GIS lattice parameters expected from symmetric XRD. Thus, the GIS alloys are not strained to match the Si lattice, although they still have an epitaxial relationship with Si. In this way, the van der Waals bonds formed at the Si/GIS interface are strong enough to establish epitaxy, yet weak enough to permit large lattice mismatch. Different surface terminations of Si(111) and different GIS lattice parameters all lead to Si(111) || GIS(001) and Si[1–10] || GIS[100] relationships—where the exact nature and limits of this phenomenon should be the subject of future study. Epitaxy of GaSe and InSe films on diverse surfaces, as discussed in the Introduction (Si(111) with As-, H-, and no termination, as well as Si(100), Si(110), GaAs(111), GaAs(001), mica, and α-Al_2_O_3_(001)), indicates that the growth mode is robust and relatively insensitive to particular substrate/epilayer bonds.

Future experiments should use much thinner 2D templates to explore the prospect of nucleating for example GaAs on the van der Waals GIS(001) planes, which lack dangling bonds. If successful, GIS alloys could act as epitaxial lift-off templates for several II–VI, III–V,^47,48^ and group IV semiconductor materials to dramatically reduce substrate wafer costs, as illustrated in Figure 7. In this work, InSe’s hexagonal *c* corresponds to a cubic *a* of 5.653 Å, making it lattice-matched with GaAs (5.654 Å). Moreover, GIS growth in the miscibility gap (hexagonal *c* of 16.150 Å; cubic *a* of 5.416 Å; Vegard’s law x of 0.621) occurred near the Si-lattice-matched (5.429 Å) x composition of 0.587, making GIS alloys of broad interest for epitaxy.

**Figure 7:**
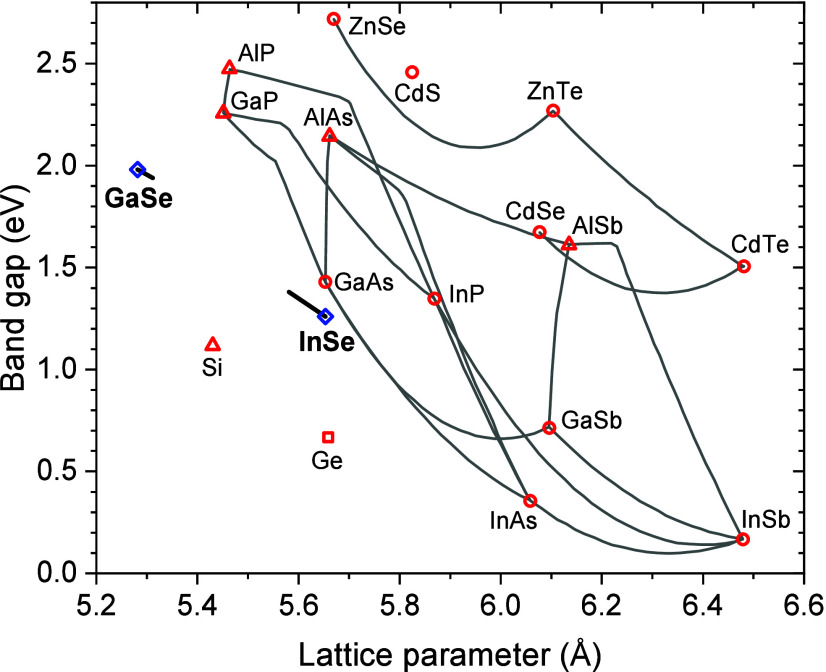
Band gaps and cubic lattice parameters of technologically important covalent semiconductors, with PL-derived band gaps and cubic equivalent lattice parameters of GIS alloys grown in this work shown as blue diamonds and black lines, illustrating the potential for semiconductor growth on GIS templates for exfoliation and substrate reuse.

## V Summary

In this work, van der Waals epitaxy of a range of 2D Ga_*x*_In_1–*x*_Se alloy compositions has been demonstrated for the first time. The Si(111) substrate led to epitaxy of GIS(001) alloys with x values of 0, 0.062, 0.164, 0.680, 0.894, and 1, as verified by XRD pole figure and TEM analysis. The films had tunable band-edge emission from 1.26 eV for InSe to 1.98 eV for GaSe, where a Se capping layer prevented oxidation even after prolonged storage. Epitaxy occurred despite lattice mismatch, where InSe was 4.1% too large and GaSe was 2.8% too small. These GIS alloys have a technologically advantageous combination of tunable band gap and photoluminescence with relaxed lattice-matching requirements and rotational registry to the substrate. This combination makes Ga_*x*_In_1–*x*_Se alloys suitable for a wide range of applications, including solar energy conversion, in tandem photovoltaic and photoelectrochemical cells, and as epitaxial lift-off layers for semiconductor materials grown on a variety of single-crystal substrates.

## Supplementary Material



## Notes

Si wafer preparation method; calibration of Se and In source temperatures to deposition rates; correlation between Ga/(Ga+In) set point and final film XRF; XRD scans with maximized and minimized substrate peak intensity; comparison of symmetric XRD and GIXRD with a 2° incidence angle for x = 0, 0.062, 0.164, 0.894, and 1; {103} pole figures for x = 0.062 and 0.894; TEM micrographs for x = 0.894; comparison of symmetric XRD and GIXRD with a 2° incidence angle for x = 1 and reduced thickness to eliminate polycrystalline GIS (PDF)
