# Supplementary material for: Lattice-Mismatched van der Waals Epitaxy and Photoluminescence of Two-Dimensional Ga x In1–x Se Alloys on Si(111)
Source: Cryst Growth Des. 2026 Jun 30;26(14):5346–54. doi: 10.1021/acs.cgd.6c00184 (PMC13383640; doi:10.1021/acs.cgd.6c00184)
Supplement: Supplementary file 1 [file cg6c00184_si_001.pdf]

Supporting Information for **Lattice-mismatched van der Waals epitaxy and photoluminescence of two-dimensional $Ga_xIn_{1-x}Se$ alloys on Si(111)**

Christopher P. Muzzillo,[1]* William E. McMahon,[1] Andriy Zakutayev,[1] and Andrew G. Norman[1]

[1]*National Laboratory of the Rockies, 15013 Denver West Pkwy, Golden, CO 80401, USA*

*Email: christopher.muzzillo@nlr.gov.

The Si wafers were 'RCA cleaned,' with the final oxide etch delayed until just before loading into the vacuum: 10 min in a solution of 5 parts de-ionized (DI) water, 1 part 29% NH_4OH, and 1 part 30% H_2O_2 held at 75 °C, DI water rinse, 1 min in 1% HF solution, DI water rinse, 10 min in a solution of 6 parts DI water, 1 part 37% HCl, and 1 part 30% H_2O_2 held at 75 °C, DI water rinse, N_2 blow dry, storage in fluoroware in an N_2 purge desiccator, DI water rinse, 1 min in 1% HF solution, DI water rinse, N_2 blow dry, and immediate transfer into the load lock. After loading, the Si wafers were ramped at 30 °C/min to 680 °C and held for 10 min, which should lead to complete desorption of the H.

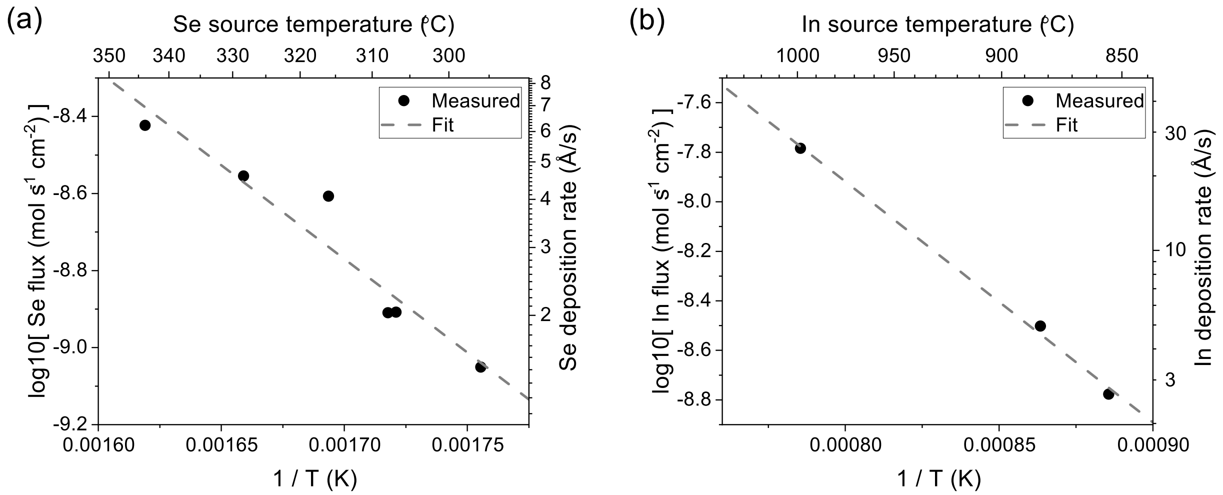

Fig. S1. Log10 of molar flux from profilometry as a function of inverse source temperature for Se (a) and In (b).

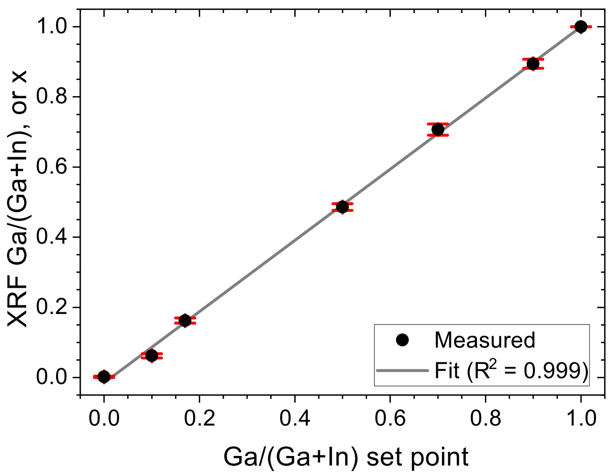

Fig. S2. XRF Ga/(Ga+In), or x, of the final film as a function of Ga/(Ga+In) set point.

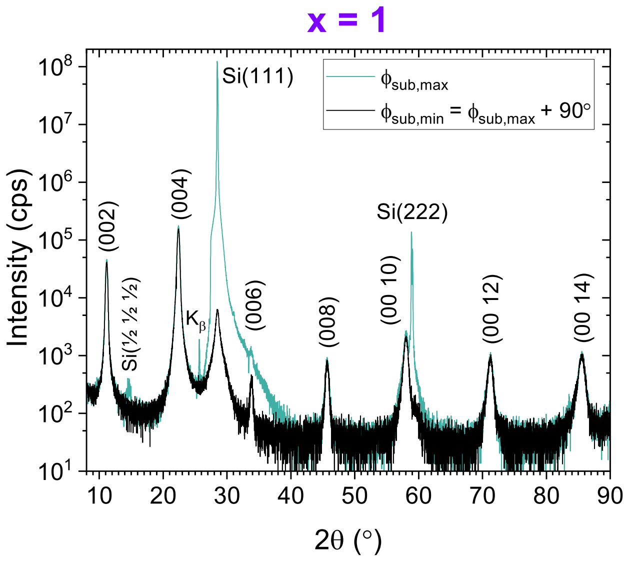

Fig. S3. Symmetric XRD patterns for Si/GaSe collected at the maximum substrate peak intensity φ (teal), and φ rotated 90° (black), showing how wafer miscut was utilized to avoid obscuring GIS film peaks.

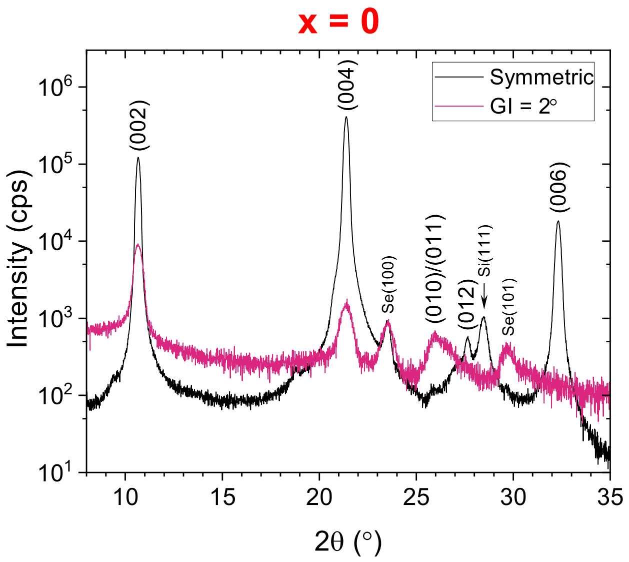

Fig. S4. Symmetric (black) and GIXRD with an incidence angle of 2° for x = 0, showing polycrystalline GIS and crystalline Se at the surface, and a uniform GIS lattice parameter throughout the GIS film thickness.

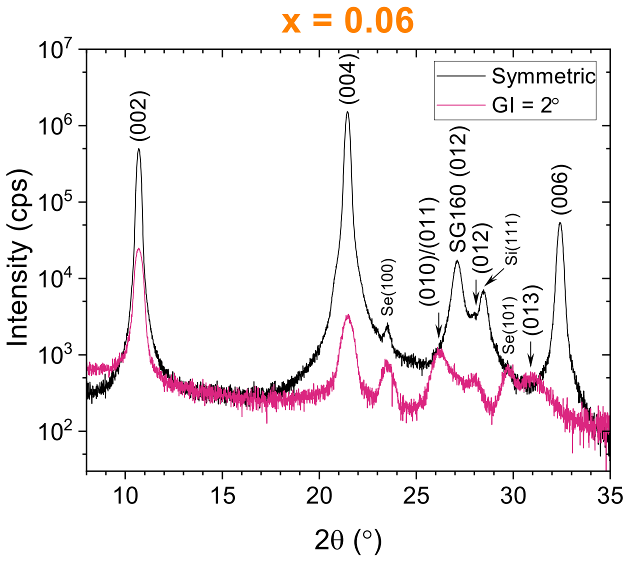

Fig. S5. Symmetric (black) and GIXRD with an incidence angle of 2° for x = 0.06, showing polycrystalline GIS and crystalline Se at the surface, and a uniform GIS lattice parameter throughout the GIS film thickness.

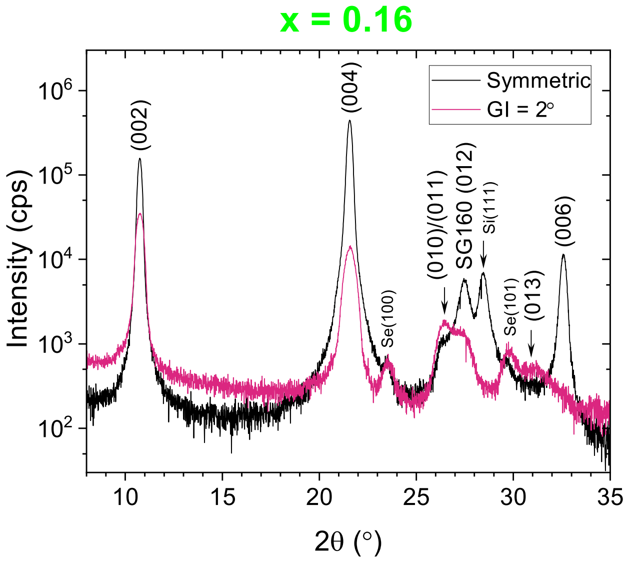

Fig. S6. Symmetric (black) and GIXRD with an incidence angle of 2° for x = 0.16, showing polycrystalline GIS and crystalline Se at the surface, and a uniform GIS lattice parameter throughout the GIS film thickness.

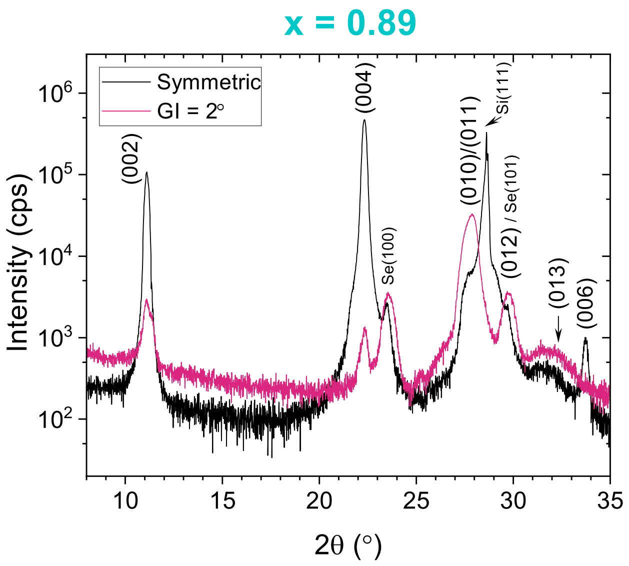

Fig. S7. Symmetric (black) and GIXRD with an incidence angle of 2° for x = 0.89, showing polycrystalline GIS and more crystalline Se at the surface, and a uniform GIS lattice parameter throughout the GIS film thickness.

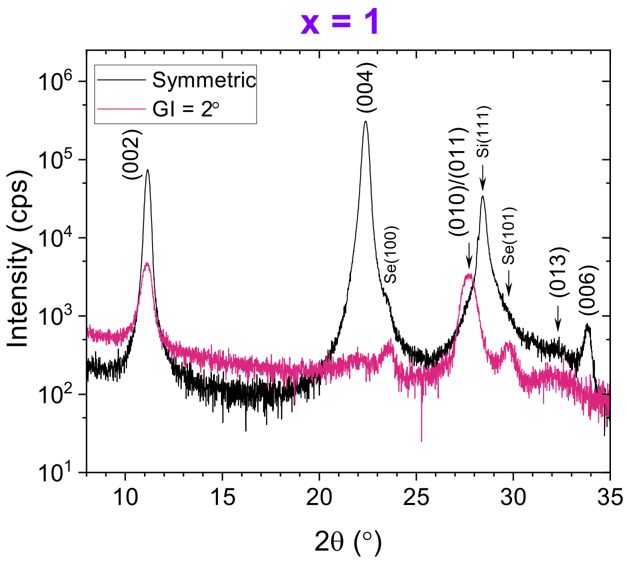

Fig. S8. Symmetric (black) and GIXRD with an incidence angle of 2° for x = 1, showing polycrystalline GIS and crystalline Se at the surface, and a uniform GIS lattice parameter throughout the GIS film thickness.

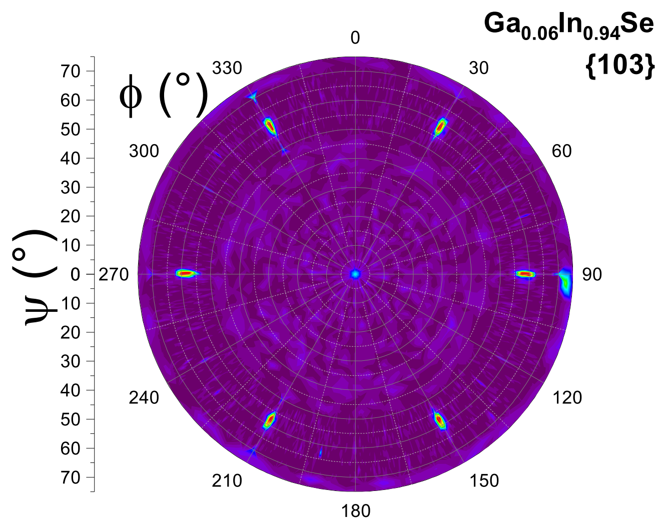

Fig. S9. {103} pole figure on an epitaxial films of $Ga_{0.06}In_{0.94}Se$ on a Si(111) substrate with $\phi = 0°$ parallel to Si[2-1-1], showing excellent rotational order in the plane of the substrate.

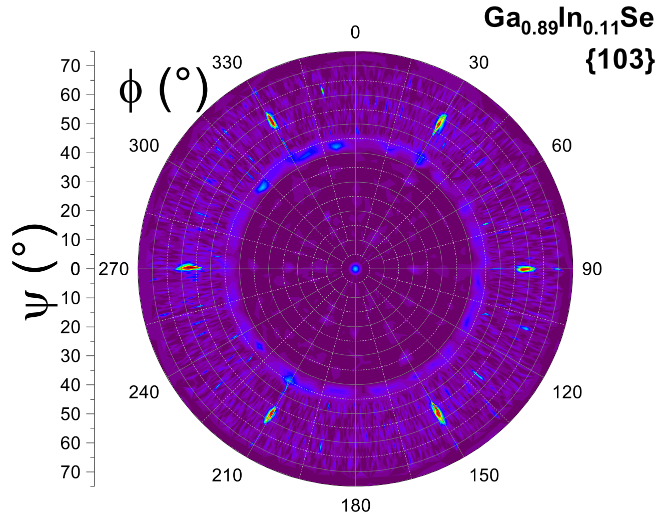

Fig. S10. {103} pole figure on an epitaxial films of $Ga_{0.89}In_{0.11}Se$ on a Si(111) substrate with $\phi = 0°$ parallel to Si[2-1-1], showing excellent rotational order in the plane of the substrate.

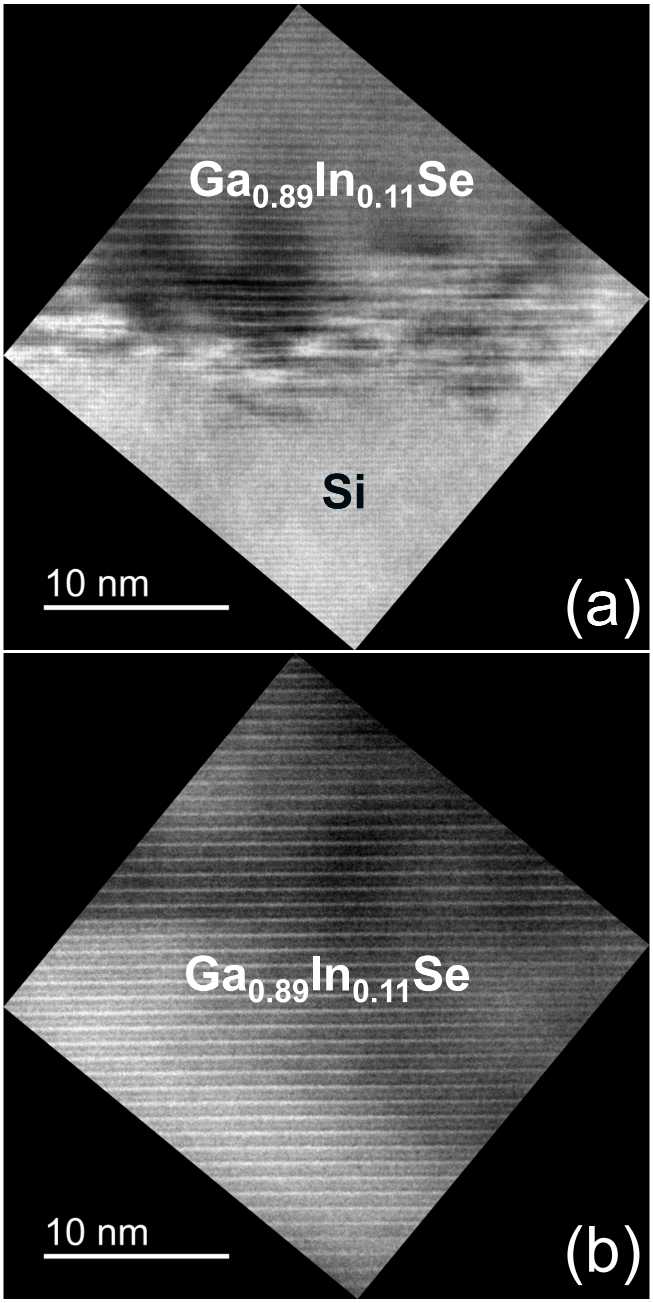

Fig. S11. HRTEM micrographs of a FIB-prepared cross-section from a $Si/Ga_{0.89}In_{0.11}Se$ (x = 0.894) sample (a) at the substrate/epilayer interface, showing lattice-nonmatched epitaxy, and (b) 10s of nanometers away from the interface, showing enhanced uniformity of the 2D layers.

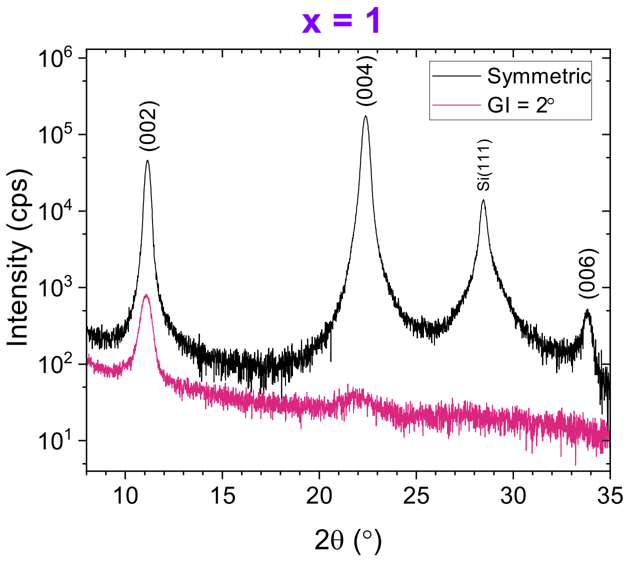

Fig. S12. Symmetric (black) and GIXRD with an incidence angle of 2° for x = 1, showing *no* polycrystalline GIS and crystalline Se at the surface (unlike Fig. S4 – S8), and a uniform GIS lattice parameter throughout the GIS film thickness.
